# MHCBN 4.0: A database of MHC/TAP binding peptides and T-cell epitopes

**DOI:** 10.1186/1756-0500-2-61

**Published:** 2009-04-20

**Authors:** Sneh Lata, Manoj Bhasin, Gajendra PS Raghava

**Affiliations:** 1 Bioinformatics Center, Institute of Microbial Technology, Sector 39A, Chandigarh, India; 2 BIDMC Genomic Center, Harvard Medical School, 238 HIM Building, 77 Avenue Louis Pasteur, Boston, MA 02115, USA

## Abstract

**Background:**

Many databases housing the information about MHC binders and non-binders have been developed in the past to help the scientific community working in the field of immunology, immune-informatics or vaccine design. As the information about these MHC binding and non-binding peptides continues to grow with the time and there is a need to keep the databases updated. So, in order to provide the immunological fraternity with the most recent information we need to maintain and update our database regularly. In this paper, we describe the updated version of 4.0 of the database MHCBN.

**Findings:**

MHCBN is a comprehensive database comprising over 25,857 peptide sequences (1053 TAP binding peptides), whose binding affinity with either MHC or TAP molecules has been assayed experimentally. It is a manually curated database where entries are collected & compiled from published literature and existing immunological public databases. MHCBN has a number of web-based tools for the analysis and retrieval of information like mapping of antigenic regions, creation of allele specific dataset, BLAST search, various diseases associated with MHC alleles etc. Further, all entries are hyper linked to major databases like SWISS-PROT, PDB etc. to provide the information beyond the scope of MHCBN. The latest version 4.0 of MHCBN has 6080 more entries than previously published version 1.1.

**Conclusion:**

MHCBN database updating is meant to facilitate immunologist in understanding the immune system and provide them the latest information. We feel that our database will complement the existing databases in serving scientific community.

## Findings

The information about peptides binding to Major Histocompatibility Complex (MHC) or Transport associated Antigen Processing (TAP) molecules and their ability to activate T-cell response has a pivotal role to play in the development of computational methods for subunit vaccine designing. The key issue in subunit vaccine design is to search an antigenic region in an antigen [1] that has the potential to stimulate T cells, and hence are called T cell epitopes. Fortunately a large amount of data is available in literature about such peptides. There was a dire need to collect and compile the information about these peptides at one single place. In the past, a number of databases have been developed to serve the scientific community. The databases like SYFPEITHI [2], FIMM [3] and HIV database [4] are modest in size and provide precise information. MHCPEP [5] is a widely used comprehensive database of MHC binding peptides but has not been updated since 1998 and doesn't include the tools for data extraction and analysis.

In order to overcome the limitations of MHCPEP and to provide information about a large number of peptides, two databases MHCBN [6] and AntiJen/JenPep [7-9] were developed. Recently a database IEDB [10-12] has been developed, which is a comprehensive knowledge centre, a repository of immune epitopes. These immunological database/resources are serving the scientific community working in the field of immunology, immune-informatics or vaccine design [13-18]. MHCBN has a number of unique features and is heavily used (~1000 hits per day) and has been cited (~70 times) by immunologists since it was created (in 2002). It is a well-maintained database with regular updates and growing continuously in term of entries and new features. In this paper, we describe the recent and updated version 4.0 of MHCBN.

### Database description and availability

**Figure 1:**
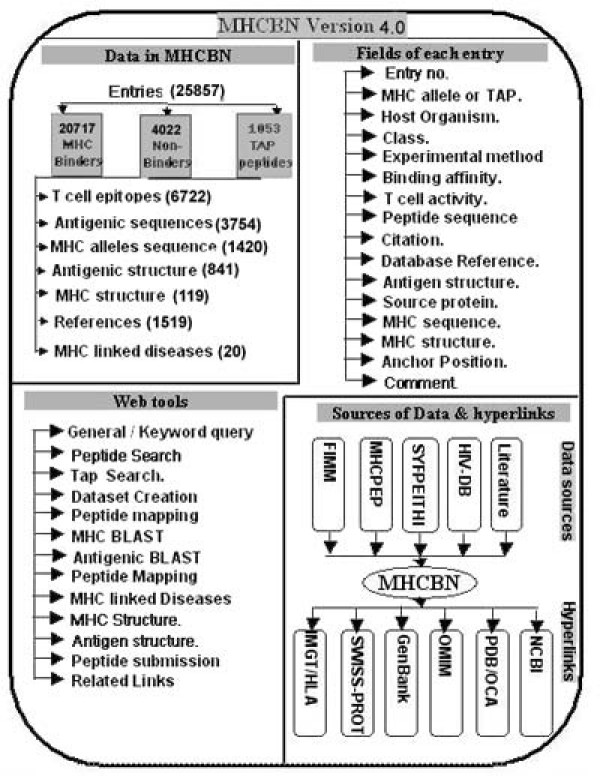
**Detailed architecture of MHCBN database**.

The updated version of database MHCBN is freely available at  or  (mirror site). PostgresSQL relational database management system (RDBMS) has been used for storing, retrieving and managing the data. All the scripts have been written using programming language PERL; CGIperl has been used for common gateway interface and Pgperl for accessing information from PostgreSQL. The database MHCBN has been developed and launched on SUN machine T1000 under Solaris 10.0 environment using Apache httpd server. The detailed architecture of the database is depicted in figure 1. Following is brief description of data available at MHCBN 4.0.

#### MHC binders

MHCBN provides comprehensive information of about 20720 MHC binders (Tables 1, 2, 3 and 4). Information about the MHC binding peptide includes, peptide sequence, source protein, binding affinity with MHC allele, T-cell activity etc. All entries are manually annotated.

**Table 1:** Statistics of MHCBN data specific for various Human MHC Class-1 alleles

**MHC Allele**	**MHC Binders**	**MHC Non-Binders**	**T-cell epitopes**
**HLA-A**	4781	497	1437

**HLA-B**	2778	183	904

**HLA-Cw**	137	15	53

**HLA-G + HLA-E**	25	0	0

**TAP**	643		

**Table 2:** Statistics of MHCBN data specific for various Human MHC Class-2 alleles

**MHC Allele**	**MHC Binders**	**MHC Non-Binders**	**T-cell epitopes**
**HLA-DR**	7486	2759	1606

**HLA-DQ**	779	161	147

**HLA-DP**	123	9	84

**Table 3:** Statistics of MHCBN data specific for mouse alleles

**MHC Allele**	**MHC Binders**	**MHC Non-Binders**	**T-cell epitopes**
	1599	82	738

	691	22	416

**H-2K**	895	53	485

**H-2D**	497	73	399

**H-2L**	129	12	64

**H-2B**	79	0	79

**H-2Q**	76	0	18

**H-2s**	31	0	31

**H-2A**	63	0	18

**other mhc-alleles**	45	--	

**TAP**	60		

**Table 4:** Statistics of MHCBN data for other organism's alleles

**MHC Allele**	**MHC Binders**	**MHC Non-Binders**	**T-cell epitopes**
**RT1(rat)**	161	151	33

**Patr(chimpanzee)**	21	0	21

**mamu(monkey)**	203	59	87

**Other mhc-alleles(Saoe, ELA-A)**	6	0	4+2

**TAP**	350		

#### MHC non-binders

One of the major challenges in the field of immune-informatics is to develop methods for predicting MHC binders in an antigenic sequence. Both negative and positive examples play equally important role in the development of a prediction method [19]. Therefore, this database database also harbours information about more than 4000 non-binders (Table 1, 2, 3, 4), which may prove beneficial to the immunological fraternity. Even the large comprehensive database IEDB maintains great information about the negative peptide or non-binders.

#### T-cell Epitopes

The database consists of more than 6700 T-cell epitopes that may by either T_helper _or CTL epitopes (Table 1, 2, 3, 4). Comprehensive information is provided about each epitope that includes its sequence, MHC binding allele, reference etc. Our collection also, has epitopes, whose MHC binding allele is not known.

#### TAP binders

Another unique feature of MHCBN is that it provides complete information about TAP binding peptides. Current version of MHCBN has more than 1000 TAP binders (Table 1, 2, 3, 4). These TAP binders are important for understanding endogenous antigen processing and for developing method to predicting TAP binders [15-17].

#### MHC Sequence and Structure

MHCBN maintains complete amino acid sequences of those MHC alleles whose binding peptide is available in the database. This may be fruitful to understand the relationship between the MHC sequence and its binding peptides. In addition, MHCBN also maintains tertiary structure information of those MHC alleles, whose structures have been solved and available in Protein Databank (PDB). Structural information can be exploited for docking of MHC binders in MHC grooves.

#### Antigen Sequence and Structure

This database consists of amino acid sequences of more than 3700 antigens. These antigens are sources of the peptides maintained in our database. These antigens are important to understand conservation in and around binding region (i.e. binding regions and amino acids around MHC binders). In addition, the database also has structures of around 840 antigens. These antigenic structures are useful to understand structure of a MHC binder in its native protein.

#### MHC-linked diseases

The database also provides information about diseases associated with various MHC alleles (e.g. autoimmune diseases). MHC allele responsible for a particular disease can be easily obtained by specifying the name of the disease or vice versa. The field is linked to OMIM database for more detailed information about the particular disease.

### Description of web tools

MHCBN provides a number of online web tools that allow the users to retrieve and analyze the information. These web tools have been designed to facilitate the user in retrieving information from the database. Following are the main tools provided in MHCBN:

#### General query

General query option allows the user to perform a keyword search on any field of the database and to extract the detailed information. The keyword can be a peptide sequence, its source protein, MHC allele, published references etc. A user can get specific and precise information by selecting appropriate values of the MHC allele, MHC class, host organism, binding strength and T cell activity in the form. User can also restrict the display of information by selecting only required fields.

#### Peptide search

This option allows user to search their peptide against peptides/binders in MHCBN. In addition to identical search, server also allows searching of binders, which have few residue mismatches with query peptide. It has a number of options including search against a specific class of MHC binders, binders of an organism, T-cell epitopes.

#### Searching TAP binders

A user can search TAP binders in our database using various options. The data binding to TAP transporter will be useful in understanding the process of endogenous antigen processing. It is also beneficial in analysis of TAP-binding peptides and development of better prediction methods.

#### Dataset creation

One of the major goals behind the development of MHCBN is to provide a comprehensive source of data for the development of new and more accurate computational methods useful in subunit vaccine design. This interactive tool thus comes to aid for the creation of allele-specific datasets. MHCBN allows user to create dataset from database using their own conditions like creation of dataset of binders for a particular MHC allele, for specific binding affinity (high, moderate, low).

#### Peptide mapping

This tool allows mapping of MHC binders, TAP binders and T-cell epitopes available in the database on a query protein sequence. Therefore, users can locate experimentally proven antigenic and non-antigenic regions in their query sequence. A user can map a MHC binder, non-binder, or T cell epitopes of specific organism on the query sequence by selecting appropriate value of host organism and type of peptides for mapping in the peptide mapping form.

#### Online data submission

The database has a facility for online submission of MHC binding, non-binding peptides and T-cell epitopes. This will help us in maintaining the comprehensive database up-to-date. In order to maintain the quality, the database team cross-checks the submitted entries before inclusion in the database.

#### BLAST search against MHC/antigenic sequences

This tool allows BLAST [20] search of query protein sequence against database of MHC alleles or antigenic peptide sequences. The BLAST search is useful in determining whether the query sequence belongs to MHC molecules or not.

### MHC-linked disease search

The updated version of MHCBN database also provides information about diseases associated with various MHC alleles (autoimmune disease). One can simply enter the name of the disease and click submit button to find out the MHC alleles associated with that disease or enter the name of the allele and find the diseases associated with that particular MHC allele. For example, MHC alleles responsible for rheumatoid arthritis can be easily obtained by specifying the name of disease or vice versa. This field is linked to OMIM database for more detailed information about a particular disease.

## Conclusion

The aim of developing and maintaining MHCBN database is to facilitate immunologists in understanding the immune system. Researchers working in the field of immunology particularly in subunit vaccine designing are heavily using this database. Developers are using the data from MHCBN for testing and training their methods. Recently, a mega project has been initiated at NIAID for creating a repository of epitopes. The IEDB is a major resource of epitopes and has more entries than any other existing database in this filed. The question arises then is whether it's worth to maintain a database like MHCBN which has got moderate size in comparison to IEDB. Authors feel that it is worth maintaining MHCBN as it has a number of entries and web tools, which are not available at IEDB. MHCBN has a total of 16035 peptide entries that can not found in IEDB. Similarly, the IEDB has 21029 entries (19883 unique peptides) distinct from MHCBN. It is also worth to have more than one comprehensive database in a field in order to provide alternate sources of information in case of unavailability or failures of one database. Thus, this database will complement the existing databases in serving scientific community. Following are major unique features not available in other resources. We are continuously extracting MHC binders and T-cell epitopes from literature since 2002 and curating data manually, thus we have a lot of epitopes/binders not available in IEDB. In addition, this database maintains information about MHC non-binders and TAP binders too, which are not maintained in any existing database. One of the powerful and unique features of MHCBN is integration of web tools. Integration of BLAST allows user to search their query sequence against known antigens and MHC alleles. This allows user to know whether their sequence is antigenic or not. Similarly, peptide mapping tool allows user to scan/identify known experimentally proved antigenic regions in their protein sequence. This is important to identify the therapeutic potential of a protein. In addition we provide information about MHC linked diseases too. In summary, MHCBN has a number of web tools, which allow extracting useful information from this database. We are working to integrate more tools and adding other type of information like comprehensive information of existing subunit vaccines (e.g. failed, in clinical phase).

## Competing interests

The authors declare that they have no competing interests.

## Authors' contributions

MB originally developed the databases. SL updated the database and put >3000 entries as well as modified the front end of the database. SL also prepared the manuscript. GPSR conceived the project, coordinated it and refined the manuscript drafted by SL.
